# O Impacto Clínico e Econômico do Atraso na Terapia de Reperfusão: Evidências do Mundo Real

**DOI:** 10.36660/abc.20230650

**Published:** 2024-04-22

**Authors:** Silvio Gioppatto, Paulo Sousa Prado, Mariana Araújo Lima Elias, Verônica Homem de Carvalho, Caio Resende da Costa Paiva, Gustavo de Almeida Alexim, Ricardo Torres Bispo Reis, Ana Claudia Cavalcante Nogueira, Alexandre Anderson de Sousa Munhoz Soares, Wilson Nadruz, Luiz Sergio F. de Carvalho, Andrei C. Sposito

**Affiliations:** 1 Universidade Estadual de Campinas Departamento de Cardiologia Campinas SP Brasil Universidade Estadual de Campinas (Unicamp) - Departamento de Cardiologia, Campinas, SP – Brasil; 2 Universidade de Brasília Brasília DF Brasil Universidade de Brasília, Brasília, DF – Brasil; 3 Hospital de Base do Distrito Federal Brasília DF Brasil Hospital de Base do Distrito Federal, Brasília, DF – Brasil; 4 Instituto Aramari Apo Brasília DF Brasil Instituto Aramari Apo, Brasília, DF – Brasil

**Keywords:** Síndrome Coronariana Aguda, Infarto do Miocárdio com Supradesnível do Segmento ST, Intervenção Coronária Percutânea, Reperfusão

## Abstract

**Fundamento:**

A terapia de reperfusão precoce é reconhecida como a abordagem mais eficaz para reduzir as taxas de letalidade de casos em pacientes com infarto do miocárdio com supradesnivelamento do segmento ST (IAMCSST).

**Objetivo:**

Estimar as consequências clínicas e econômicas do atraso da reperfusão em pacientes com IAMCSST.

**Métodos:**

O presente estudo de coorte retrospectivo avaliou as taxas de mortalidade e as despesas totais decorrentes do atraso na terapia de reperfusão em 2.622 indivíduos com IAMCSST. Os custos de cuidados hospitalares e perda de produtividade por morte ou incapacidade foram estimados sob a perspectiva do Sistema Único de Saúde indexado em dólares internacionais (Int$) ajustados pela paridade do poder de compra. Foi considerado estatisticamente significativo p < 0,05.

**Resultados:**

Cada hora adicional de atraso na terapia de reperfusão foi associada a um aumento de 6,2% (intervalo de confiança de 95%: 0,3% a 11,8%, p = 0,032) no risco de mortalidade hospitalar. As despesas gerais foram 45% maiores entre os indivíduos que receberam tratamento após 9 horas em comparação com aqueles que foram tratados nas primeiras 3 horas, impulsionados principalmente pelos custos hospitalares (p = 0,005). Um modelo de regressão linear multivariada indicou que para cada 3 horas de atraso na trombólise, houve um aumento nos custos hospitalares de Int$ 497 ± 286 (p = 0,003).

**Conclusões:**

Os achados do nosso estudo oferecem mais evidências que enfatizam o papel crucial da terapia de reperfusão imediata no salvamento de vidas e na preservação dos recursos de saúde pública. Estes resultados enfatizam a necessidade urgente de implementação de uma rede para gerir casos de IAMCSST.

## Graphical abstract

**Figura Central:**
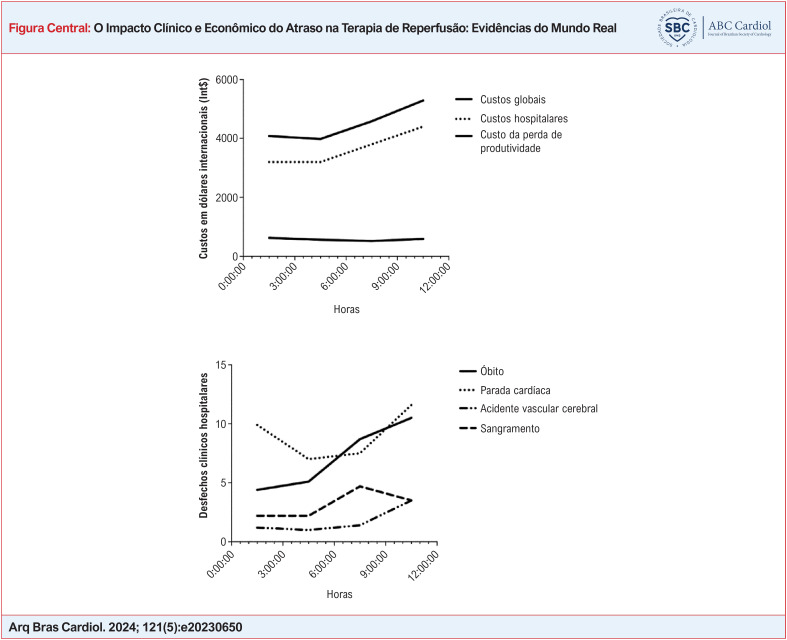
Impacto Clínico e Econômico do Atraso na Terapia de Reperfusão: Evidências do Mundo Real

## Introdução

O infarto do miocárdio com supradesnivelamento do segmento ST (IAMCSST) é uma das principais causas de mortalidade em todo o mundo.^1^ A terapia de reperfusão precoce é amplamente reconhecida como a estratégia mais eficaz para reduzir a letalidade em pacientes com IAMCSST.^1^ Estudos têm demonstrado que os pacientes que não recebem terapia de reperfusão têm um risco 3 a 4 vezes maior de morte em comparação com aqueles que recebem.^2^

Embora as evidências apoiem os benefícios semelhantes da intervenção coronária percutânea (ICP) primária e da trombólise farmacológica seguida de ICP,^3^ a prática atual é transferir os pacientes com IAMCSST para centros com capacidade para ICP o mais rápido possível. Infelizmente, esta abordagem tem resultado em um número significativo de casos de IAMCSST não reperfundidos (até 40%) devido à distribuição desigual de centros com capacidade para ICP.^4 , 5^

Esforços têm sido feitos para resolver a questão dos casos de IAMCSST não reperfundidos através do estabelecimento de redes de IAMCSST, que provaram ser bem-sucedidas em garantir o acesso universal à terapia de reperfusão.^6 , 7^ No entanto, a divulgação dessas estratégias permanece limitada, destacando a necessidade de maior conscientização entre os prestadores de cuidados de saúde à sua relação custo-efetividade.

Os custos diretos e indiretos das estratégias de reperfusão têm sido adequadamente abordados em indivíduos que recebem ICP primária ou fibrinólise em comparação com aqueles que não recebem.^8 , 9^ No entanto, o impacto econômico da terapia de reperfusão atrasada (por hora de atraso) permanece incerto. Portanto, nosso estudo visou medir os custos diretos e indiretos associados à terapia de reperfusão atrasada em indivíduos com IAMCSST. A presente análise oferecerá uma melhor compreensão do possível aumento das taxas de mortalidade e das despesas associadas ao atraso na terapia de reperfusão.

## Métodos e desenho do estudo

### População do estudo

O presente estudo de coorte retrospectivo incluiu todos os indivíduos com IAMCSST que receberam trombólise farmacológica e/ou foram submetidos a um cateterismo cardíaco na Rede de Saúde Pública do Distrito Federal (Brasília, DF, Brasil) entre janeiro de 2011 e dezembro de 2019. Os critérios de inclusão do estudo exigiram que os pacientes atendessem às seguintes condições: (i) elevação do segmento ST de pelo menos 1 mm (plano frontal) ou 2 mm (plano horizontal) em 2 derivações contíguas ou bloqueio de ramo esquerdo recentemente presumido ou bloqueio de ramo direito; (ii) necrose miocárdica demonstrada por aumento de pelo menos um valor acima do limite de referência do percentil 99 de CK-MB (25 U/L) e troponina I (0,04 ng/mL), seguido de declínio subsequente em ambos; (iii) trombólise farmacológica administrada dentro de 24 horas do início dos sintomas ou ICP primária dentro de 48 horas do início dos sintomas; e (iv) cateterismo cardíaco durante a internação índice.

Foram identificados indivíduos (n = 2.622) com IAMCSST que atenderam aos critérios de inclusão. O presente estudo foi conduzido de acordo com a Declaração de Helsinque e recebeu aprovação do Comitê de Ética em Pesquisa do Instituto de Gestão Estratégica do Distrito Federal (IGEDPF) (número de aprovação do protocolo do estudo [CAAE] 28530919.0.1001.8153). Por se tratar de um estudo retrospectivo, o Comitê de Ética em Pesquisa aprovou a dispensa do consentimento informado dos participantes, desde que os dados fossem coletados anonimamente.

### Desfechos clínicos (desfechos primários e secundários)

O desfecho primário foi composto pelos custos globais, uma soma dos custos hospitalares mais o custo da perda de produtividade (CPP) devido à morte ou incapacidade. Embora os custos hospitalares incluam o impacto de procedimentos de alto custo, como diálise, cirurgia de revascularização do miocárdio (CRM), ICP, contrapulsação com balão intra-aórtico e tempo gasto em unidades de terapia intensiva (detalhes na Tabela Suplementar S1), o CPP estima o impacto da perda da capacidade de trabalho na população economicamente ativa. Os desfechos secundários incluíram desfechos clínicos, como incidência de morte por todas as causas, infarto do miocárdio recorrente, CRM, parada cardíaca, fibrilação atrial aguda, acidente vascular cerebral e sangramento grave. Os desfechos clínicos foram observados exclusivamente durante a internação hospitalar.

### Dados clínicos

Avaliamos sistemas de registros eletrônicos de saúde e coletamos dados como demografia, informações sobre apresentação do IAMCSST, histórico médico passado, terapias hospitalares, informações sobre procedimentos de alto custo, medicamentos na alta hospitalar e desfechos.

### Dados angiográficos

A gravidade anatômica, a extensão da doença aterosclerótica coronariana, os tratamentos angiográficos e a função ventricular esquerda foram analisados por meio de relatórios escritos. A classificação da gravidade da estenose adotada foi uma redução do lúmen arterial > 70% para vasos epicárdicos e > 50% no tronco da artéria coronária esquerda. A doença multiarterial foi caracterizada por 3 ou mais vasos epicárdicos principais com estenose do lúmen arterial > 70% ou com envolvimento do tronco principal esquerdo com estenose > 50%.^10^ A função ventricular esquerda foi definida como preservada na presença de contratilidade normal e a disfunção foi definida como a presença de hipocinesia ou acinesia.

### Dados de mortalidade

A ocorrência de óbitos foi determinada por meio de consulta ao Sistema de Informação sobre Mortalidade (SIM/SUS) do Ministério da Saúde. A codificação das certidões de óbito no SIM é realizada utilizando um sistema de codificação automatizado. Todos os óbitos exigem uma declaração de causa (certidão de óbito) emitida por um médico.

### Avaliação de custos

Os custos diretos incluíram os cuidados hospitalares, incluindo exames, procedimentos e utilização de unidades de cuidados intensivos. Consideramos a perspectiva do Sistema Único de Saúde (SUS) como pagador. Os valores reembolsados para itens de custos são padronizados em todo o país com base na tabela de preços do SUS (descrito na e-Tabela 1). Para os custos brasileiros, os valores monetários da tabela de preços do SUS foram obtidos em reais (R$) e posteriormente convertidos para dólares internacionais (Int$) considerando a paridade do poder de compra (fator de conversão 2,36) para o ano de 2015 (ano mediano de admissão entre os indivíduos do estudo). Esse método de extração de dados dos bancos de dados do SUS já foi descrito anteriormente.^10^

Para avaliar os custos indiretos, ou seja, os custos associados à perda de produtividade devido à morte hospitalar nesta população, avaliamos o CPP e os anos de vida produtiva perdidos (AVPP). Os AVPP representam o número de anos de perda de produtividade decorrente da impossibilidade dos indivíduos participarem do mercado de trabalho devido à sua condição.^11^ Para calcular os AVPP, primeiro distribuímos os óbitos por faixa etária. A idade média de cada grupo foi então calculada e subtraída da idade de aposentadoria.^12^ O número de óbitos em cada grupo etário foi multiplicado pelo número de anos restantes para atingir a idade de aposentadoria. Neste caso, utilizamos a idade de aposentadoria de 65 anos. A soma desses produtos fornece o número total de anos de vida produtiva potencial perdidos devido à síndrome coronariana aguda prematura.

O CPP é calculado multiplicando-se os AVPP pela soma da renda total estimada desde a idade da morte precoce até a idade da aposentadoria em indivíduos com síndrome coronariana aguda prematura. A renda foi baseada no salário médio brasileiro no período corrigido pela taxa de desemprego. Representa a perda de produtividade em valor econômico.^13 , 14^ O salário médio mensal entre 2011 e 2019 foi de R$ 1.000. (Int$ 366,30) para mulheres e R$ 1.664,00 (Int$ 609,52) para homens. A taxa de desemprego neste período foi de aproximadamente 7%. Nessas análises, não foi possível avaliar o impacto dos eventos clínicos ocorridos após a alta.

### Análise estatística

Foram divididos 4 grupos de acordo com o tempo entre o início dos sintomas e a trombólise química: dentro de 3 horas após o início dos sintomas, entre 3 e 6 horas, entre 6 e 9 horas e após 9 horas. As análises primárias incluíram todos os indivíduos tratados por abordagem farmacoinvasiva, ICP de resgate ou ICP eletiva. As análises de sensibilidade foram realizadas excluindo indivíduos tratados por ICP de resgate ou ICP eletiva. A distribuição das variáveis e sua normalidade foram verificadas por meio de histogramas, gráficos de dispersão e o teste de Kolmogorov-Smirnov. Para comparação entre os grupos, utilizamos o teste qui-quadrado para variáveis categóricas, análise de variância (ANOVA) unidirecional para variáveis contínuas com distribuição normal e teste de Kruskal-Wallis para variáveis contínuas com distribuição não paramétrica. Quando uma diferença estatisticamente significativa foi detectada usando ANOVA ou teste de Kruskal-Wallis, realizamos comparações post-hoc entre pares entre as médias dos grupos usando o teste de Bonferroni ou Dunn, respectivamente. Para analisar a incidência de óbito durante o acompanhamento clínico, foram construídos modelos de regressão logística binária e modelos de regressão linear para avaliar o impacto do tempo desde o início dos sintomas até a trombólise química. Antes de realizar essas análises de regressão linear, avaliamos os seguintes pressupostos: linearidade, normalidade multivariada, ausência de multicolinearidade e de autocorrelação, homoscedasticidade e nível de medição. Empregamos um modelo de regressão logística bivariada para avaliar o risco de morte hospitalar por hora de atraso desde o início dos sintomas até a terapia de reperfusão. Posteriormente, foi utilizado um modelo de regressão logística multivariada para identificar os preditores independentes de óbito hospitalar por meio de um processo *stepwise* (método para frente). Os dados foram apresentados como média ± desvio padrão para dados com distribuição normal e como mediana (intervalo interquartil) para dados com distribuição não normal. As variáveis categóricas foram apresentadas como número absoluto (%) e comparadas pelo teste do qui-quadrado ou teste exato de Fisher quando apropriado. O valor de p < 0,05 foi considerado estatisticamente significativo. As análises estatísticas foram realizadas utilizando R Studio v.1.1.463, linguagem R versão 4.0.1 para Mac.

## Resultados

O estudo incluiu 2.622 participantes, dos quais 944 (36%) receberam trombólise farmacológica dentro de 3 horas do início dos sintomas, 1.146 (43,7%) entre 3 e 6 horas, 358 (13,7%) entre 6 e 9 horas e 172 (6,6%) após 9 horas. A maioria dos pacientes tinha histórico de hipertensão e o grupo tratado após 9 horas apresentava uma proporção significativamente maior de indivíduos com diabetes tipo 2 prévio (p < 0,001). Outras comorbidades relatadas na Tabela 1 foram distribuídas uniformemente entre os grupos. Além disso, os critérios eletrocardiográficos para reperfusão após trombólise foram menos frequentes no grupo tratado após 9 horas (p < 0,001).

**Tabela 1:** Características dos pacientes incluídos

	Tempo desde o início dos sintomas até a terapia de reperfusão no IAMCSST				p
	< 3 h	3-6 h	6-9 h	> 9 h	
N	944	1148	358	172	
**Dados demográficos e apresentação clínica**					
Sexo masculino (%)	585 (61,9)	689 (60,0)	229 (63,9)	105 (61,0)	0,841
Idade, anos (média [DP])	60,54 (9,26)	62,72 (7,64)	63,90 (9,51)	64,08 (8,84)	0,121
**Comorbidades**					
Diabetes mellitus prévio (%)	239 (25,3)	351 (30,6)	129 (36,0)	77 (44,8)	<0,001
Obesidade prévia (%)	191 (20,2)	210 (18,3)	70 (19,6)	40 (23,3)	0,402
Hipertensão prévia (%)	533 (56,5)	723 (63,0)	215 (60,1)	107 (62,2)	0,023
Histórico familiar prévio de DAC (%)	195 (20,7)	246 (21,4)	76 (21,2)	32 (18,6)	0,850
Abuso prévio de drogas ilícitas (%)	30 (3,2)	43 (3,7)	21 (5,9)	9 (5,2)	0,119
Dislipidemia prévia (%)	474 (50,2)	575 (50,1)	164 (45,8)	80 (46,5)	0,411
IM prévio (%)	89 (9,4)	111 (9,7)	27 (7,5)	16 (9,3)	0,678
AVC prévio (%)	29 (3,1)	48 (4,2)	17 (4,7)	10 (5,8)	0,237
ICP prévia (%)	49 (5,2)	51 (4,4)	12 (3,4)	10 (5,8)	0,454
DAP prévia (%)	40 (4,2)	51 (4,4)	16 (4,5)	7 (4,1)	0,992
DRC prévia (%)	56 (5,9)	82 (7,1)	24 (6,7)	17 (9,9)	0,272
CRM prévia (%)	12 (1,3)	24 (2,1)	6 (1,7)	5 (2,9)	0,351
Tabagismo prévio (%)	625 (66,2)	724 (63,1)	217 (60,6)	99 (57,6)	0,072
IMC (média [DP])	27,02 (4,54)	26,86 (4,53)	26,61 (4,67)	27,39 (4,75)	0,245
Creatinina plasmática (mediana [IIQ])	0,91 [0,76; 1,10]	0,90 [0,75; 1,10]	0,90 [0,74; 1,12]	0,90 [0,74; 1,20]	0,855
**Eletrocardiograma**					
BRE (%)	1 (0,1)	2 (0,2)	5 (1,4)	4 (2,3)	<0,001
Redução do segmento ST ≥ 50% após terapia de reperfusão (%)	647 (68,5)	860 (74,9)	264 (73,7)	96 (55,8)	<0,001
**Cuidados cardiovasculares agudos**					
Tempo desde os sintomas até o hospital primário (média [DP])	64,54 (34,26)	154,77 (70,64)	277,90 (114,51)	455,25 (228,80)	<0,001
Tempo sintomas-agulha (mediana [IIQ])	120 [90; 150]	240 [210; 290]	410 [380; 462]	630 [570; 731]	<0,001
Tempo desde a trombólise farmacológica até o hospital terciário (mediana [IIQ])	330 [200; 540]	332 [208; 615,5]	369,5 [222; 664]	400 [245; 790]	0,001
Tempo desde a trombólise farmacológica até o cateterismo (média [DP])	1115,81 (1154,80)	1282,36 (1237,06)	1368,27 (1135,76)	1380,97 (1379,48)	<0,001
PAS na admissão (média [DP])	133,45 (27,85)	133,91 (28,18)	132,15 (27,36)	130,30 (26,74)	0,366
Frequência cardíaca na admissão (média [DP])	78,30 (17,51)	79,07 (17,26)	79,89 (17,60)	82,08 (19,39)	0,054
**Classificação de Killip (%)**					<0,001
I	902 (95,6)	1098 (95,6)	323 (90,2)	144 (83,7)	
II	36 (3,8)	43 (3,7)	34 (9,5)	24 (14,0)	
III	6 (0,6)	7 (0,6)	1 (0,3)	4 (2,3)	
**Estratégia de ICP (%)**					<0,001
Farmacoinvasiva	594 (62,9)	792 (69,0)	243 (67,9)	95 (55,2)	
ICP de resgate	332 (35,2)	324 (28,2)	100 (27,9)	73 (42,4)	
ICP eletiva	18 (1,9)	32 (2,8)	15 (4,2)	4 (2,3)	
Número de stents convencionais (média [DP])	0,92 (0,79)	0,85 (0,70)	0,89 (0,78)	0,74 (0,70)	0,008
Número de SF (mediana [IIQ])	0,08 (0,33)	0,09 (0,37)	0,06 (0,31)	0,15 (0,44)	0,015
Inibidores da glicoproteína IIb/IIIa (%)	45 (4,8)	57 (5,0)	24 (6,7)	18 (10,5)	0,045
Dobutamina (%)	4 (0,4)	12 (1,0)	11 (3,1)	10 (5,8)	0,025
Adenosina durante o cateterismo (%)	47 (5,0)	41 (3,6)	6 (1,7)	11 (6,4)	0,015
FEVE no terceiro dia (%, média [DP])	52,04 (12,90)	52,03 (13,44)	52,03 (12,51)	51,50 (13,00)	0,966
GRACE (morte hospitalar) (média [DP])	115,08 (39,34)	118,98 (39,32)	123,06 (43,91)	125,25 (41,90)	<0,001
**Lesões da artéria coronária e resultados de ICP**					
Doença principal esquerda (%)	10 (1,1)	15 (1,3)	6 (1,7)	4 (2,3)	0,542
Doença de 3 vasos (%)	182 (19,3)	244 (21,3)	78 (21,8)	43 (25,0)	0,320
Doença de 2 vasos (%)	263 (27,9)	362 (31,5)	103 (28,8)	56 (32,6)	0,248
Doença uniarterial (%)	434 (46,0)	457 (39,8)	147 (41,1)	59 (34,3)	0,005
**Grau de fluxo TIMI pós-ICP (%)**					0,006
0	167 (17,7)	204 (17,8)	59 (16,5)	49 (28,5)	
1	11 (1,2)	13 (1,1)	6 (1,7)	3 (1,7)	
2	130 (13,8)	143 (12,5)	49 (13,7)	32 (18,6)	
3	636 (67,4)	788 (68,6)	244 (68,2)	88 (51,2)	
**Grau de blush pós-ICP (%)**					0,036
0	361 (38,2)	411 (35,8)	138 (38,5)	89 (51,7)	
1	69 (7,3)	86 (7,5)	26 (7,3)	9 (5,2)	
2	49 (5,2)	67 (5,8)	17 (4,7)	4 (2,3)	
3	465 (49,3)	584 (50,9)	177 (49,4)	70 (40,7)	
**Laboratoriais**					
Pico de troponina (mUI/dL, mediana [IIQ])	5929 [2703; 10471]	6354 [3076; 10973]	5576 [2886; 9798]	5951 [3268; 11703]	0,311
Hemoglobina (g/dL, média [DP])	14,64 (1,73)	14,48 (1,81)	14,19 (1,96)	14,51 (2,06)	0,001
Glicemia na admissão (média [DP])	145,02 (75,75)	147,23 (73,02)	148,98 (74,28)	165,49 (85,45)	0,012
HbA1c (%, média [DP])	6,11 (1,63)	6,32 (1,77)	6,57 (2,10)	6,78 (2,34)	<0,001

AVC: acidente vascular cerebral; BRE: bloqueio de ramo esquerdo; CRM: cirurgia de revascularização do miocárdio; DAC: doença arterial coronariana; DAP: doença arterial periférica; DP: desvio padrão; DRC: doença renal crônica; IAMCSST: infarto do miocárdio com supradesnivelamento do segmento ST; ICP: intervenção coronária percutânea; IIQ: intervalo interquartil; IM: infarto do miocárdio; IMC: índice de massa corporal; PAS: pressão arterial sistólica; SF: stents farmacológicos.

A Figura Central ilustra os principais resultados deste estudo. A estratégia farmacoinvasiva foi administrada à maioria dos indivíduos em todos os grupos, e o grupo que recebeu tratamento após 9 horas teve maior frequência de cateterismo de resgate (conforme mostrado na Tabela 1 ). Os pacientes que receberam trombólise atrasada tiveram um escore GRACE médio mais alto (p < 0,001), enquanto tempos de tratamento mais longos foram associados a níveis elevados de Hb1Ac (p < 0,001). Os níveis de pico de troponina não diferiram significativamente entre os grupos.

A Tabela 2 demonstra que o grupo que recebeu tratamento após 9 horas teve maior incidência de morte hospitalar (p = 0,001), infarto do miocárdio recorrente (p = 0,026), parada cardíaca (p = 0,040) e acidente vascular cerebral (p = 0,049). O sangramento maior foi mais prevalente no grupo tratado entre 6 e 9 horas, seguido pelo grupo tratado após 9 horas e depois pelos grupos tratados dentro de 3 horas e entre 3 e 6 horas (p = 0,040). Em um modelo bivariado (conforme mostrado na Tabela 3 ), cada hora adicional desde o início dos sintomas até a terapia de reperfusão foi associada a um risco 8,1% (intervalo de confiança de 95%: 2,5% a 13,2%, p = 0,003) maior de morte hospitalar (129 eventos).

**Tabela 2:** Desfechos

		Tempo desde o início dos sintomas até a trombólise no IAMCSST				p
		< 3 h	3-6 h	6-9 h	> 9 h	
**Desfechos clínicos**						
	Mortes hospitalares (%)	42 (4,4)	58 (5,1)	31 (8,7)	18 (10,5)	0,001
	CRM intra-hospitalar após IAMCSST (%)	36 (3,8)	41 (3,6)	17 (4,7)	3 (1,7)	0,386
	Tempo de internação (dias, mediana [IIQ])	4,0 [3,0; 5,0]	4,0 [3,0; 5,0]	4,0 [3,0; 6,0]	4,0 [2,75; 5,0]	0,958
	IM recorrente intra-hospitalar (%)	16 (1,7)	16 (1,4)	6 (1,7)	8 (4,7)	0,026
	Parada cardíaca (%)	93 (9,9)	80 (7,0)	27 (7,5)	20 (11,6)	0,040
	Fibrilação atrial aguda (%)	37 (3,9)	36 (3,1)	16 (4,5)	3 (1,7)	0,321
	Acidente vascular cerebral (%)	11 (1,2)	11 (1,0)	5 (1,4)	6 (3,5)	0,049
	Sangramento maior (%)	21 (2,2)	25 (2,2)	17 (4,7)	6 (3,5)	0,040
**Desfechos relacionados a custos**						
	Custos globais (Int$, mediana [IIQ])	4081 [1800; 7700]	3981 [1800; 7581]	4581 [1800; 8181]	5290 [2400; 8810]	0,023
	Custo dos cuidados hospitalares (Int$, mediana [IIQ])	3200 [1800; 6200]	3200 [1800; 6200]	3800 [1800; 7700]	4400 [2400; 8450]	0,005
	Custo da perda de produtividade (Int$, média [DP])	627 (872)	562 (815)	520 (751)	590 (769)	0,282

CRM: cirurgia de revascularização do miocárdio; DP: desvio padrão; IAMCSST: infarto do miocárdio com supradesnivelamento do segmento ST; IIQ: intervalo interquartil; IM: infarto do miocárdio.

* Os custos diretos foram calculados a partir dos valores descritos na e-Tabela 1 utilizando dados do Brasil obtidos do DATASUS (SIH/SUS e SIGTAP), o sistema de processamento de dados do Ministério da Saúde.

Foi empregado um modelo de regressão logística multivariada *stepwise* para determinar os preditores independentes de morte hospitalar, conforme mostrado na Tabela 3 . A análise revelou que o tempo decorrido desde o início dos sintomas até a terapia de reperfusão foi um preditor independente de morte hospitalar. Especificamente, cada hora adicional de atraso na reperfusão foi associada a um aumento de 6,2% (intervalo de confiança de 95%: 0,3% a 11,8%, p = 0,032) no risco de morte hospitalar. Outros preditores independentes significativos de morte hospitalar incluíram a necessidade de reperfusão de resgate, diabetes, hipertensão, doença multiarterial, fração de ejeção do ventrículo esquerdo (FEVE) e escore GRACE.

**Tabela 3:** Modelos de regressão logística com morte hospitalar (129 eventos) como variável dependente

(modelo bivariado)	OR	Inferior	Superior	p
Tempo desde o início dos sintomas até a terapia de reperfusão (por atraso de 1 hora)	1,081	1,025	1,132	0,003027
**(modelo multivariado stepwise)**	**OR**	**Inferior**	**Superior**	**p**
Tempo desde o início dos sintomas até a terapia de reperfusão (por atraso de 1 hora)	1,062	1,003	1,118	0,0322
Reperfusão de resgate versus abordagem farmacoinvasiva	6,650	4,580	9,822	<0,0001
Diabetes mellitus prévio	1,613	1,126	2,307	0,0089
Hipertensão prévia	1,833	1,220	2,817	0,0044
Tabagismo prévio	0,726	0,510	1,036	0,0763
Número de SF	0,711	0,352	1,228	0,2774
Doença uniarterial	0,372	0,241	0,559	<0,0001
FEVE (por redução de 10%)	1,312	1,165	1,463	<0,0001
Escore GRACE para morte hospitalar (por aumento de 10 pontos)	1,384	1,328	1,443	<0,0001

FEVE: fração de ejeção do ventrículo esquerdo; OR: odds ratio; SF: stents farmacológicos.

Os custos globais foram 45% maiores entre os indivíduos tratados após 9 horas do que entre os indivíduos tratados dentro de 3 horas, um impacto impulsionado principalmente pelos cuidados hospitalares (p = 0,005). Porém, o CPP entre os indivíduos tratados após 9 horas não foi diferente entre os grupos. Por fim, um modelo de regressão linear multivariada mostrou que cada atraso de 3 horas na realização da trombólise estava associado a um aumento nos custos de cuidados hospitalares de Int$ 497 ± 286 ( Tabela 4 ; p = 0,003). O modelo multivariado foi ajustado por reperfusão de resgate versus abordagem farmacoinvasiva, diabetes mellitus prévio, hipertensão prévia, tabagismo prévio, número de stents farmacológicos durante ICP, doença uniarterial, FEVE e escore GRACE para morte hospitalar, atingindo um R^2^ de 0,452.

**Tabela 4:** Modelos de regressão linear para custos hospitalares

(modelo bivariado)	beta	DP	p
Tempo desde o início dos sintomas até a terapia de reperfusão (por atraso de 3 horas	603,91	303,37	0,00015
**(modelo multivariado stepwise)** *	**beta**	**DP**	**p**
Tempo desde o início dos sintomas até a terapia de reperfusão (por atraso de 3 horas	496,62	286,10	0,00335

DP: desvio padrão.

* Modelo multivariado ajustado por: reperfusão de resgate versus abordagem farmacoinvasiva, diabetes mellitus prévio, hipertensão prévia, tabagismo prévio, número de stents farmacológicos durante a intervenção coronária percutânea, doença uniarterial, fração de ejeção do ventrículo esquerdo e escore GRACE para morte hospitalar. Modelo R2 = 0,452.

As análises de sensibilidade foram realizadas avaliando exclusivamente os 1.724 pacientes tratados com abordagem farmacoinvasiva. A Tabela Suplementar 2 mostra que 594 receberam trombólise química dentro de 3 horas após o início dos sintomas, 792 entre 3 e 6 horas, 243 entre 6 e 9 horas e 95 após 9 horas. A frequência de comorbidades entre os grupos foi homogênea em comparação com as análises globais (n = 2.622). As mortes hospitalares foram menos frequentes naqueles tratados < 3 horas (1,9%) ou entre 3 e 6 horas (2,3%), em comparação com aqueles entre 6 e 9 horas (4,5%) ou > 9 horas (7,3%; p = 0,021). Os custos globais foram 50% maiores entre os indivíduos tratados após 9 horas do que entre os pacientes tratados dentro de 3 horas, um impacto impulsionado principalmente pelos cuidados hospitalares (p = 0,023).

## Discussão

As doenças cardíacas são um fardo importante nos Estados Unidos, resultando em um custo anual de aproximadamente 229 bilhões de dólares e 0,7 milhão de mortes (1 em cada 5) todos os anos.^15 , 16^ Globalmente, as doenças cardíacas são responsáveis por 17,9 milhões de mortes anualmente.^17^ Estes números persistentes levaram os prestadores de cuidados de saúde públicos e privados a tomar medidas. Nosso estudo mostra que antecipar a reperfusão pode não apenas reduzir a mortalidade, mas também diminuir o custo geral associado ao IAMCSST. Para cada hora de atraso, a mortalidade hospitalar aumentou em 6%, e para cada 3 horas, o custo aumentou em cerca de Int$ 500. Nossos achados reforçam os benefícios clínicos da terapia de reperfusão imediata e destacam as potenciais economias de custos que podem ser alcançadas por meio de estratégias bem planejadas para facilitar tratamento mais rápido.

A reperfusão oportuna é crucial no tratamento do IAMCSST, pois qualquer atraso pode ter consequências graves, incluindo aumento da incapacidade e mortalidade. Consistente com nossos achados, um grande estudo retrospectivo construído a partir de registros médicos dinamarqueses de base populacional revelou que cada hora de atraso na terapia de reperfusão resulta em um aumento de 10% na mortalidade.^18^ Complementamos essa informação mostrando que as consequências da reperfusão atrasada ou inadequada também pode ter impactos financeiros significativos no sistema de saúde.

Além disso, os pacientes e suas famílias podem potencialmente enfrentar impactos financeiros significativos devido ao atraso na reperfusão. Por exemplo, um estudo transversal realizado no Sri Lanka revelou que 40% dos sobreviventes de infarto do miocárdio que não receberam terapia de reperfusão tiveram que procurar assistência financeira para as suas despesas diretas.^19^ Além disso, o estudo verificou que 5% dos pacientes perderam seus empregos; 29% tinham atividade física limitada enquanto permaneciam empregados; 40% tinham restrições de tempo de emprego; 15,4% solicitaram empréstimos; 7,8% venderam sua propriedade; 19,1% sofreram perda de renda; e 33,8% tiveram que reduzir suas despesas habituais.^19^ No Brasil, 38% das mortes cardiovasculares ocorrem em indivíduos em idade produtiva, o que causa uma perda produtiva equivalente a 15% do custo total associado às doenças cardiovasculares.^14^

De fato, as doenças cardiovasculares são particularmente preocupantes nos países de renda baixa e média, onde se estima que ocorram 80% de todas as mortes cardiovasculares.^20^ Nesses países, o IAMCSST tende a afetar indivíduos mais jovens em idade ativa, resultando em significativas consequências econômicas diretas e indiretas. Além disso, as projeções sugerem que a perda econômica acumulada devido às doenças cardiovasculares nos países de baixa e média renda, entre 2011 e 2025, atingirá aproximadamente 3,76 trilhões de dólares estadunidenses.^20^

Embora a FEVE, a classe Killip e os níveis de troponina tenham sido semelhantes entre os grupos de acordo com o tempo desde o início dos sintomas até a trombólise no IAMCSST, os pacientes tratados após 9 horas necessitaram de ICP de resgate com mais frequência, o que foi um dos principais impulsionadores de piores resultados. Além disso, como a ICP de resgate normalmente exige uma transferência hospitalar demorada, os pacientes tratados após 9 horas também apresentaram menor grau de *blush* miocárdico e de fluxo TIMI, levando ao aumento do uso de inibidores da glicoproteína IIb/IIIa, adenosina e dobutamina ( Tabela 1 ). Esses achados podem estar subjacentes à ocorrência de desfechos clínicos, como acidente vascular cerebral, sangramento maior e parada cardíaca, o que pode ter contribuído para a maior taxa de mortalidade em pacientes tratados posteriormente. Portanto, dado que a FEVE e a troponina foram semelhantes entre os grupos, é mais provável que as mortes tenham sido causadas por fatores não cardíacos ou indiretos.

Em seu conjunto, esses achados destacam a necessidade de estratégias bem planeadas para facilitar terapia de reperfusão mais rápida. Essas estratégias podem incluir melhorias no transporte e triagem de pacientes, implementação de protocolos de tratamento eficazes e utilização de tecnologias avançadas para agilizar o processo de terapia de reperfusão. Ao desenvolver e implementar tais estratégias, os prestadores de cuidados de saúde podem garantir que os pacientes recebam tratamento rápido e eficaz, o que pode levar a melhores desfechos e custos mais baixos.

Na interpretação dos nossos achados, é importante reconhecer certas limitações. Embora tenhamos conseguido ilustrar as implicações de custo da terapia de reperfusão atrasada, nosso estudo não teve poder estatístico para estimar o custo por hora de atraso. Além disso, não tínhamos acesso a alguns dados relativos à ampla gama de impactos financeiros que podem ocorrer nas famílias de pacientes com IAMCSST, o que nos teria permitido gerar uma estimativa mais abrangente do custo total associado ao atraso da reperfusão. Embora os resultados tenham sido consistentes em toda a coorte do estudo, o tamanho limitado da amostra de pacientes que receberam terapia farmacoinvasiva tornou a análise de custos pouco confiável neste grupo específico de pacientes. Também é válido notar que os nossos métodos para estimar os custos indiretos têm uma limitação, uma vez que não conseguimos captar incapacidades clínicas importantes a longo prazo ou eventos clínicos que ocorreram após a alta; portanto, depende apenas de mortes hospitalares.

## Conclusão

Resumindo, nossos resultados fornecem mais evidências que apoiam o papel crítico da terapia de reperfusão imediata na preservação de vidas e dos recursos de saúde pública. A ausência de um sistema eficaz para o manejo de casos de IAMCSST gera tanto custos financeiros diretos, como despesas hospitalares, quanto custos indiretos associados à perda de anos produtivos devido à morte prematura ou à redução da capacidade de trabalho.

## Data Availability

Todas as solicitações de dados brutos e analisados e materiais relacionados, excluindo códigos de programação, serão analisadas pelo departamento jurídico da Clarity Healthcare Intelligence para verificar se a solicitação está sujeita a quaisquer obrigações de propriedade intelectual ou confidencialidade. Solicitações de dados relacionados ao paciente podem ser consideradas mediante solicitação. Quaisquer dados e materiais que possam ser compartilhados serão divulgados por meio de um Contrato de Transferência de Materiais.

## *Material suplementar

Para informação adicional do Material Suplementar 1, por favor, clique aqui. https://abccardiol.org/supplementary-material/2024/12105/2023-0650_supplementary_material.pdf

Para informação adicional do Material Suplementar 2, por favor, clique aqui. https://abccardiol.org/supplementary-material/2024/12105/2023-0650_supplementary_checklist.pdf
