# Supplementary material for: O Impacto Clínico e Econômico do Atraso na Terapia de Reperfusão: Evidências do Mundo Real
Source: Arq Bras Cardiol. 2024 Apr 22;121(5):e20230650. [Article in Portuguese] doi: 10.36660/abc.20230650 (PMC11081405; doi:10.36660/abc.20230650)
Supplement: Translation

# Translation: The Clinical and Economic Impact of Delayed Reperfusion Therapy: Real-World Evidence

**DOI:** 10.36660/abc.20230650i

**Authors:** Silvio Gioppatto, Paulo Sousa Prado, Mariana Araújo Lima Elias, Verônica Homem de Carvalho, Caio Resende da Costa Paiva, Gustavo de Almeida Alexim, Ricardo Torres Bispo Reis, Ana Claudia Cavalcante Nogueira, Alexandre Anderson de Sousa Munhoz Soares, Wilson Nadruz, Luiz Sergio F. de Carvalho, Andrei C. Sposito

**Affiliations:** 1 Campinas SP Brazil Universidade Estadual de Campinas (Unicamp) - Departamento de Cardiologia, Campinas, SP – Brazil; 2 Brasília DF Brazil Universidade de Brasília, Brasília, DF – Brazil; 3 Brasília DF Brazil Hospital de Base do Distrito Federal, Brasília, DF – Brazil; 4 Brasília DF Brazil Instituto Aramari Apo, Brasília, DF – Brazil

**Keywords:** Acute Coronary Syndrome, ST Elevation Myocardial Infarction, Percutaneous Coronary Intervention, Reperfusion

## Abstract

**Background:**

Early reperfusion therapy is acknowledged as the most effective approach for reducing case fatality rates in patients with ST-segment elevation myocardial infarction (STEMI).

**Objective:**

Estimate the clinical and economic consequences of delaying reperfusion in patients with STEMI.

**Methods:**

This retrospective cohort study evaluated mortality rates and the total expenses incurred by delaying reperfusion therapy among 2622 individuals with STEMI. Costs of in-hospital care and lost productivity due to death or disability were estimated from the perspective of the Brazilian Unified Health System indexed in international dollars (Int$) adjusted by purchase power parity. A p < 0.05 was considered statistically significant.

**Results:**

Each additional hour of delay in reperfusion therapy was associated with a 6.2% increase (95% CI: 0.3% to 11.8%, p = 0.032) in the risk of in-hospital mortality. The overall expenses were 45% higher among individuals who received treatment after 9 hours compared to those who were treated within the first 3 hours, primarily driven by in-hospital costs (p = 0.005). A multivariate linear regression model indicated that for every 3-hour delay in thrombolysis, there was an increase in in-hospital costs of Int$497 ± 286 (p = 0.003).

**Conclusions:**

The findings of our study offer further evidence that emphasizes the crucial role of prompt reperfusion therapy in saving lives and preserving public health resources. These results underscore the urgent need for implementing a network to manage STEMI cases.

## Introduction

ST-elevation myocardial infarction (STEMI) is a leading cause of mortality worldwide.^1^Early reperfusion therapy is widely recognized as the most effective strategy for reducing lethality in patients with STEMI.^1^Studies have demonstrated that patients who do not receive reperfusion therapy are at a 3 to 4 times higher risk of death compared to those who do.^2^

Although evidence supports similar benefits of primary percutaneous coronary intervention (PCI) and pharmacologic thrombolysis followed by PCI,^3^the current practice is to transfer patients with STEMI to PCI-capable centers as quickly as possible. Unfortunately, this approach has resulted in a significant number of non-reperfused STEMI cases (up to 40%) due to the uneven distribution of PCI-capable centers.^4 , 5^

Efforts have been made to address the issue of non-reperfused STEMI cases through the establishment of STEMI networks, which have proven to be successful in ensuring universal access to reperfusion therapy.^6 , 7^However, the dissemination of these strategies remains limited, highlighting the need for greater awareness among healthcare providers regarding their cost-effectiveness.

Direct and indirect costs of reperfusion strategies have been properly addressed in individuals receiving versus not receiving primary PCI or fibrinolysis.^8 , 9^However, the economic impact of delayed reperfusion therapy (per hour delayed) remains unclear. Therefore, our study aims to measure both direct and indirect costs associated with delayed reperfusion therapy in individuals with STEMI. This analysis will offer a better understanding of the possible rise in mortality rates and expenses linked to delayed reperfusion therapy.

## Study design and methods

### Study population

This retrospective cohort study included all individuals with STEMI who received pharmacological thrombolysis and/or underwent cardiac catheterization in the Public Healthcare System of the Federal District (Brasília, DF, Brazil) between January 2011 and December 2019. The study's inclusion criteria required patients to meet the following conditions: (i) ST-segment elevation of at least 1 mm (frontal plane) or 2 mm (horizontal plane) in 2 contiguous leads or a newly presumed left bundle branch block or right bundle branch block; (ii) myocardial necrosis demonstrated by an increase in at least one value above the 99^th^percentile reference limit of CK-MB (25 U/L) and troponin I (0.04 ng/mL), followed by a subsequent decline in both; (iii) pharmacological thrombolysis administered within 24 hours of symptom onset or primary PCI within 48 hours of symptom onset; and (iv) cardiac catheterization during the index hospitalization.

Individuals (n = 2,622) with STEMI who met the inclusion criteria were identified. This study was conducted in accordance with the Helsinki Declaration, and it received approval from the Institutional Ethics Review Board (IRB) of the Instituto de Gestão Estratégica do Distrito Federal (IGESDF) (study protocol approval number [CAAE] 28530919.0.1001.8153). Since this was a retrospective study, the IRB approved the waiver of participants’ informed consent as long as data was captured anonymously.

### Clinical outcomes (primary and secondary outcomes)

The primary outcome was the global costs, a sum of in-hospital costs plus the cost of lost productivity (CLP) due to death or disability. While in-hospital costs include the impact of high-cost procedures such as dialysis, coronary artery bypass graft (CABG), PCI, intra-aortic balloon counterpulsation, and time spent in intensive care units (details in Supplementary Table S1), CLP estimates the impact of lost working capacity within the economically active population. The secondary outcomes included clinical outcomes such as the incidence of all-cause death, recurrent myocardial infarction (MI), CABG, cardiac arrest, acute atrial fibrillation, stroke, and major bleeding. The clinical outcomes were observed exclusively during the in-hospital stay.

### Clinical data

We assessed electronic health record systems and collected data such as demography, information regarding STEMI presentation, past medical history, in-hospital therapies, information regarding high-cost procedures, discharge medications, and outcomes.

### Angiographic data

Anatomical severity, the extent of coronary atherosclerotic disease, angiographic treatments, and left ventricular function were analyzed by means of written reports. The classification for severity of stenosis adopted was a reduction of arterial lumen > 70% for epicardial vessels and > 50% in the left main coronary artery. Multivessel disease was characterized by 3 or more major epicardial vessels with arterial lumen stenosis > 70% or left main involvement with stenosis > 50%.^10^Left ventricular function was defined as preserved in the presence of normal contractility, and dysfunction was defined as the presence of hypokinesia or akinesia.

### Mortality data

The occurrence of deaths was determined by consulting a specific information system for mortality (SIM/SUS) from the Brazilian Health Ministry. Coding of death certificates in SIM is undertaken utilizing an automated coding system. All deaths require a declaration of cause (death certificate) issued by a physician.

### Cost assessment

Direct costs included the costs of in-hospital care, including exams, procedures, and critical care unit utilization. We considered the perspective of the Brazilian Unified Health System (SUS) as the payer. The amounts reimbursed for cost items are standardized across the country based on the SUS price list (described in e-Table 1). For Brazilian costs, the monetary values of the SUS price list were obtained in Brazilian reais (BRL) and subsequently converted to international dollars (Int$) considering the purchasing power parity (conversion factor 2.36) for the year 2015 (median admission year among study individuals). This method of extracting data from SUS databases has been previously described.^10^

To assess indirect costs, i.e., costs associated with lost productivity due to in-hospital death in this population, we evaluated the CLP and years of potential productive life lost (YPPLL). YPPLL represents the number of years of lost productivity resulting from individuals not being able to participate in the workforce due to their condition.^11^To calculate the YPPLL, we first distributed the deaths by age group. We then calculated the mean age of each group and subtracted it from the retirement age.^12^The number of deaths in each age group was multiplied by the number of years left to reach the retirement age. In this case, we used the retirement age of 65 years. The sum of these products provides the total number of years of potential productive life lost due to premature acute coronary syndrome.

CLP is calculated by multiplying YPPLL by the sum of total estimated income from the age of early death to the age of retirement in individuals with premature acute coronary syndrome. This income was based on the average Brazilian salary in the period corrected for the unemployment rate. It represents the loss of productivity in economic value.^13 , 14^The mean monthly salary between 2011 and 2019 was BRL1,000. (Int$366.30) for women, and BRL1,664.00 (Int$609.52) for men. The unemployment rate in this period was around 7%. In these analyses, we could not evaluate the impact of clinical events occurring after discharge.

### Statistical analyses

Four groups were segregated according to the time between symptoms onset and chemical thrombolysis: within 3 hours after symptoms onset, between 3 and 6 hours, between 6 and 9 hours, and after 9 hours. Primary analyses included all subjects treated either by pharmacoinvasive approach, rescue PCI, or elective PCI. Sensitivity analyses were conducted by excluding individuals treated by rescue PCI or elective PCI. The distribution of variables and their normality were checked using histograms, scatter plots, and the Kolmogorov-Smirnov test. For comparison between the groups, we used the chi-square test for categorical variables, one-way analysis of variance (ANOVA) for continuous variables with normal distribution, and Kruskal-Wallis's test for continuous variables with a non-parametric distribution. When a statistically significant difference was detected using ANOVA or the Kruskal-Wallis test, we conducted post-hoc pairwise comparisons between group means using the Bonferroni or Dunn test, respectively. To analyze the incidence of death during clinical follow-up, binary logistic regression models and linear regression models were constructed to evaluate the impact of time from symptoms onset to chemical thrombolysis. Prior to conducting these linear regression analyses, we evaluated the following assumptions: linearity, multivariate normality, the absence of multicollinearity and autocorrelation, homoscedasticity, and measurement level. We employed a bivariate logistic regression model to evaluate the risk of in-hospital death per hour of delay from symptom onset to reperfusion therapy. Subsequently, a multivariate logistic regression model was utilized to identify the independent predictors of in-hospital death using a stepwise process (forward method). Data were presented as mean ± standard deviation for normally distributed data and as median (interquartile range) for non-normally distributed data. Categorical variables were presented as the absolute number (%) and were compared by chi-square test or Fisher'sexact test when appropriate. The value of p < 0.05 was considered statistically significant. Statistical analyses were performed using R Studio v.1.1.463, R language version 4.0.1 for Mac.

## Results

The study comprised 2,622 participants, out of which 944 (36%) received pharmacological thrombolysis within 3 hours of symptom onset, 1146 (43.7%) between 3 and 6 hours, 358 (13.7%) between 6 and 9 hours, and 172 (6.6%) after 9 hours. The majority of subjects had a history of hypertension, and the group treated after 9 hours had a significantly higher proportion of subjects with prior type 2 diabetes (p < 0.001). Other comorbidities reported in Table 1 were evenly distributed across the groups. Moreover, EKG criteria for reperfusion following thrombolysis were less frequent in the group treated after 9 hours (p < 0.001).

**Table 1:** Characteristics of enrolled individuals

	Time from symptoms onset to reperfusion therapy in STEMI				p
	< 3 h	3-6 h	6-9 h	> 9 h	
N	944	1148	358	172	
**Demography and clinical presentation**					
Male (%)	585 (61.9)	689 (60.0)	229 (63.9)	105 (61.0)	0.841
Age, years (mean [SD])	60.54 (9.26)	62.72 (7.64)	63.90 (9.51)	64.08 (8.84)	0.121
**Comorbidities**					
Prior diabetes mellitus (%)	239 (25.3)	351 (30.6)	129 (36.0)	77 (44.8)	<0.001
Prior obesity (%)	191 (20.2)	210 (18.3)	70 (19.6)	40 (23.3)	0.402
Prior hypertension (%)	533 (56.5)	723 (63.0)	215 (60.1)	107 (62.2)	0.023
Prior family history of CAD (%)	195 (20.7)	246 (21.4)	76 (21.2)	32 (18.6)	0.850
Prior illicit drug abuse (%)	30 (3.2)	43 (3.7)	21 (5.9)	9 (5.2)	0.119
Prior dyslipidemia (%)	474 (50.2)	575 (50.1)	164 (45.8)	80 (46.5)	0.411
Prior MI (%)	89 (9.4)	111 (9.7)	27 (7.5)	16 (9.3)	0.678
Prior stroke (%)	29 (3.1)	48 (4.2)	17 (4.7)	10 (5.8)	0.237
Prior PCI (%)	49 (5.2)	51 (4.4)	12 (3.4)	10 (5.8)	0.454
Prior PAD (%)	40 (4.2)	51 (4.4)	16 (4.5)	7 (4.1)	0.992
Prior CKD (%)	56 (5.9)	82 (7.1)	24 (6.7)	17 (9.9)	0.272
Prior CABG (%)	12 (1.3)	24 (2.1)	6 (1.7)	5 (2.9)	0.351
Prior smoking (%)	625 (66.2)	724 (63.1)	217 (60.6)	99 (57.6)	0.072
BMI (mean [SD])	27.02 (4.54)	26.86 (4.53)	26.61 (4.67)	27.39 (4.75)	0.245
Plasma creatinine (median [IQR])	0.91 [0.76; 1.10]	0.90 [0.75; 1.10]	0.90 [0.74; 1.12]	0.90 [0.74; 1.20]	0.855
**EKG**					
LBBB (%)	1 (0.1)	2 (0.2)	5 (1.4)	4 (2.3)	<0.001
ST-segment reduction ≥50% after reperfusion therapy (%)	647 (68.5)	860 (74.9)	264 (73.7)	96 (55.8)	<0.001
**Acute cardiovascular care**					
Time from symptoms to primary hospital (mean [SD])	64.54 (34.26)	154.77 (70.64)	277.90 (114.51)	455.25 (228.80)	<0.001
Symptoms-needle time (median [IQR])	120 [90; 150]	240 [210; 290]	410 [380; 462]	630 [570; 731]	<0.001
Time from pharmacological thrombolysis to tertiary hospital (median [IQR])	330 [200; 540]	332 [208; 615.5]	369.5 [222; 664]	400 [245; 790]	0.001
Time from pharmacological thrombolysis to catheterization (mean [SD])	1115.81 (1154.80)	1282.36 (1237.06)	1368.27 (1135.76)	1380.97 (1379.48)	<0.001
SBP at admission (mean [SD])	133.45 (27.85)	133.91 (28.18)	132.15 (27.36)	130.30 (26.74)	0.366
Heart rate at admission (mean [SD])	78.30 (17.51)	79.07 (17.26)	79.89 (17.60)	82.08 (19.39)	0.054
**Killip class (%)**					<0.001
I	902 (95.6)	1098 (95.6)	323 (90.2)	144 (83.7)	
II	36 (3.8)	43 (3.7)	34 (9.5)	24 (14.0)	
III	6 (0.6)	7 (0.6)	1 (0.3)	4 (2.3)	
**PCI strategy (%)**					<0.001
Pharmacoinvasive	594 (62.9)	792 (69.0)	243 (67.9)	95 (55.2)	
Rescue PCI	332 (35.2)	324 (28.2)	100 (27.9)	73 (42.4)	
Elective PCI	18 (1.9)	32 (2.8)	15 (4.2)	4 (2.3)	
Number conventional stents (mean [SD])	0.92 (0.79)	0.85 (0.70)	0.89 (0.78)	0.74 (0.70)	0.008
Number DES (median [IQR])	0.08 (0.33)	0.09 (0.37)	0.06 (0.31)	0.15 (0.44)	0.015
Glycoprotein IIb/IIIa inhibitors (%)	45 (4.8)	57 (5.0)	24 (6.7)	18 (10.5)	0.045
Dobutamine (%)	4 (0.4)	12 (1.0)	11 (3.1)	10 (5.8)	0.025
Adenosine during catheterization (%)	47 (5.0)	41 (3.6)	6 (1.7)	11 (6.4)	0.015
LVEF at the 3rd day (%, mean [SD])	52.04 (12.90)	52.03 (13.44)	52.03 (12.51)	51.50 (13.00)	0.966
GRACE (in-hospital death) (mean [SD])	115.08 (39.34)	118.98 (39.32)	123.06 (43.91)	125.25 (41.90)	<0.001
**Coronary artery lesions and PCI results**					
Left main disease (%)	10 (1.1)	15 (1.3)	6 (1.7)	4 (2.3)	0.542
3-vessel disease (%)	182 (19.3)	244 (21.3)	78 (21.8)	43 (25.0)	0.320
2-vessel disease (%)	263 (27.9)	362 (31.5)	103 (28.8)	56 (32.6)	0.248
1-vessel disease (%)	434 (46.0)	457 (39.8)	147 (41.1)	59 (34.3)	0.005
**TIMI flow grade post-PCI (%)**					0.006
0	167 (17.7)	204 (17.8)	59 (16.5)	49 (28.5)	
1	11 (1.2)	13 (1.1)	6 (1.7)	3 (1.7)	
2	130 (13.8)	143 (12.5)	49 (13.7)	32 (18.6)	
3	636 (67.4)	788 (68.6)	244 (68.2)	88 (51.2)	
**Blush grade post-PCI (%)**					0.036
0	361 (38.2)	411 (35.8)	138 (38.5)	89 (51.7)	
1	69 (7.3)	86 (7.5)	26 (7.3)	9 (5.2)	
2	49 (5.2)	67 (5.8)	17 (4.7)	4 (2.3)	
3	465 (49.3)	584 (50.9)	177 (49.4)	70 (40.7)	
**Laboratory**					
Troponin peak (mUI/dL, median [IQR])	5929 [2703; 10471]	6354 [3076; 10973]	5576 [2886; 9798]	5951 [3268; 11703]	0.311
Hemoglobin (g/dL, mean [SD])	14.64 (1.73)	14.48 (1.81)	14.19 (1.96)	14.51 (2.06)	0.001
Admission glycemia (mean [SD])	145.02 (75.75)	147.23 (73.02)	148.98 (74.28)	165.49 (85.45)	0.012
HbA1c (%, mean [SD])	6.11 (1.63)	6.32 (1.77)	6.57 (2.10)	6.78 (2.34)	<0.001

BMI: body mass index; CABG: coronary artery bypass graft; CAD, coronary artery disease; CKD: chronic kidney disease; DES: drug-eluting stents; EKG: electrocardiogram; IQR: interquartile range; LBBB: left bundle branch block; MI: myocardial infarction; PAD: peripheral artery disease; PCI: percutaneous coronary intervention; SBP: systolic blood pressure; SD: standard deviation; STEMI: ST-segment elevation myocardial infarction.

The Central Illustration illustrates the main results of this study. The pharmacoinvasive strategy was administered to the majority of subjects in all groups, and the group that received treatment after 9 hours had a higher frequency of rescue catheterization (as shown in Table 1 ). Patients who experienced delayed thrombolysis had a higher mean GRACE score (p < 0.001), while longer treatment times were associated with elevated Hb1Ac levels (p < 0.001). Peak troponin levels were not found to differ significantly between the groups.

Table 2 demonstrates that the group receiving treatment after 9 hours had a greater incidence of in-hospital death (p = 0.001), recurrent MI (p = 0.026), cardiac arrest (p = 0.040), and stroke (p = 0.049). Major bleeding was more prevalent in the group treated between 6 and 9 hours, followed by the group treated after 9 hours, and then the groups treated within 3 hours and between 3 and 6 hours (p = 0.040). In a bivariate model (as shown in Table 3 ), each additional hour from symptom onset to reperfusion therapy was associated with an 8.1% (95% CI: 2.5% to 13.2%, p = 0.003) higher risk of in-hospital death (129 events).

**Table 2:** Outcomes

		Time from symptoms onset to thrombolysis in STEMI				p
		< 3 h	3-6 h	6-9 h	> 9 h	
**Clinical outcomes**						
	In-hospital deaths (%)	42 (4.4)	58 (5.1)	31 (8.7)	18 (10.5)	0.001
	In-hospital CABG after STEMI (%)	36 (3.8)	41 (3.6)	17 (4.7)	3 (1.7)	0.386
	Length of stay (days, median [IQR])	4.0 [3.0; 5.0]	4.0 [3.0; 5.0]	4.0 [3.0; 6.0]	4.0 [2.75; 5.0]	0.958
	In-hospital recurrent MI (%)	16 (1.7)	16 (1.4)	6 (1.7)	8 (4.7)	0.026
	Cardiac arrest (%)	93 (9.9)	80 (7.0)	27 (7.5)	20 (11.6)	0.040
	Acute atrial fibrillation (%)	37 (3.9)	36 (3.1)	16 (4.5)	3 (1.7)	0.321
	Stroke (%)	11 (1.2)	11 (1.0)	5 (1.4)	6 (3.5)	0.049
	Major bleeding (%)	21 (2.2)	25 (2.2)	17 (4.7)	6 (3.5)	0.040
**Cost outcomes**						
	Global costs (Int$, median [IQR])	4081 [1800; 7700]	3981 [1800; 7581]	4581 [1800; 8181]	5290 [2400; 8810]	0.023
	Custo dos cuidados hospitalares (Int$, mediana [IIQ])	3200 [1800; 6200]	3200 [1800; 6200]	3800 [1800; 7700]	4400 [2400; 8450]	0.005
	Custo da perda de produtividade (Int$, média [DP])	627 (872)	562 (815)	520 (751)	590 (769)	0.282

CABG: coronary artery bypass graft; IQR: interquartile range; MI: myocardial infarction; SD: standard deviation; STEMI: ST-elevation myocardial infarction.

* Direct costs were calculated with values described in e-Table 1 using data for Brazil obtained from DATASUS (SIH/SUS and SIGTAP), the data processing system of the Brazilian Health Ministry.

A stepwise multivariate logistic regression model was employed to determine the independent predictors of in-hospital death, as shown in Table 3 . The analysis revealed that the time elapsed from symptom onset to reperfusion therapy was an independent predictor of in-hospital death. Specifically, each additional hour of delay in reperfusion was associated with a 6.2% (95% CI: 0.3% to 11.8%, p = 0.032) increase in the risk of in-hospital death. Other significant independent predictors of in-hospital death included the need for rescue reperfusion, diabetes, hypertension, multi-vessel disease, left ventricular ejection fraction (LVEF), and GRACE score.

**Table 3:** Logistic regression models with in-hospital death (129 events) as dependent variable

(bivariate model)	OR	Lower	Upper	p
Time from symptoms onset to reperfusion therapy (per 1-h delay)	1.081	1.025	1.132	0.003027
**(multivariate stepwise model)**	**OR**	**Lower**	**Upper**	**p**
Time from symptoms onset to reperfusion therapy (per 1-h delay)	1.062	1.003	1.118	0.0322
Rescue reperfusion versus pharmacoinvasive	6.650	4.580	9.822	<0.0001
Prior diabetes mellitus	1.613	1.126	2.307	0.0089
Prior hypertension	1.833	1.220	2.817	0.0044
Prior smoking	0.726	0.510	1.036	0.0763
Number DES	0.711	0.352	1.228	0.2774
1-vessel disease	0.372	0.241	0.559	<0.0001
LVEF (per 10% decrease)	1.312	1.165	1.463	<0.0001
GRACE score for in-hospital death (per 10-point increase)	1.384	1.328	1.443	<0.0001

DES: drug-eluting stents; LVEF: left ventricular ejection fraction; OR: odds ratio.

Global costs were 45% higher among individuals treated after 9 hours than individuals treated within 3 hours, an impact mainly driven by in-hospital care (p = 0.005). However, the CLP among individuals treated after 9 hours was not different across groups. Finally, a multivariate linear regression model showed that every 3-hour delay in performing thrombolysis was associated with an increase in in-hospital care costs by Int$497 ± 286 ( Table 4 ; p = 0.003). The multivariate model was adjusted by rescue reperfusion versus pharmacoinvasive approach, prior diabetes mellitus, prior hypertension, prior smoking, number of drug eluting stents during PCI, 1-vessel disease, LVEF, and GRACE score for in-hospital death, reaching an R^2^of 0.452.

**Table 4:** Linear regression models for in-hospital costs

(bivariate model)	beta	SD	p
Time from symptoms onset to reperfusion therapy (per 3-hour delay)	603.91	303.37	0.00015
**(multivariate stepwise model)** *	**beta**	**SD**	**p**
Time from symptoms onset to reperfusion therapy (per 3-hour delay)	496.62	286.10	0.00335

SD: standard deviation.

* Multivariate model adjusted by: rescue reperfusion versus pharmacoinvasive, prior diabetes mellitus, prior hypertension, prior smoking, number of drug-eluting stents during percutaneous coronary intervention, 1-vessel disease, left ventricular ejection fraction, and GRACE score for in-hospital death. Model R2 = 0.452.

Sensitivity analyses were conducted by evaluating exclusively the 1,724 individuals treated with a pharmacoinvasive approach. Supplementary e-Table 2 shows 594 received chemical thrombolysis within 3 hours after symptoms onset, 792 between 3 and 6 hours, 243 between 6 and 9 hours, and 95 after 9 hours. The frequency of comorbidities across groups was homogeneous compared to the global analyses (n = 2,622). In-hospital deaths were less frequent for those treated < 3 hours (1.9%) or between 3 and 6 hours (2.3%), as compared with those between 6 and 9 hours (4.5%) or > 9 hours (7.3%; p = 0.021). Global costs were 50% higher among individuals treated after 9 hours than in individuals treated within 3 hours, an impact mainly driven by in-hospital care (p = 0.023).

## Discussion

Heart disease is a major burden in the United States, resulting in an annual cost of approximately $229 billion and 0.7 million deaths (1 in 5) each year.^15 , 16^Globally, heart disease is responsible for 17.9 million deaths annually.^17^These persistent figures have prompted both public and private healthcare providers to take action. Our study shows that anticipating reperfusion can not only reduce mortality, but also lower the overall cost associated with STEMI. For every hour of delay, in-hospital mortality increases by 6%, and for every 3 hours, the cost increases by about Int$ 500. Our findings reinforce the clinical benefits of prompt reperfusion therapy and highlight the potential cost savings that can be achieved through well-planned strategies to facilitate faster treatment.

Timely reperfusion is crucial in the management of STEMI, as any delay can have severe consequences, including increased disability and mortality. Consistent with our findings, a large retrospective study built on population-based Danish medical registries revealed that each hour of delay in reperfusion therapy results in a 10% increase in mortality.^18^We complemented this information by showing that the consequences of delayed or inadequate reperfusion can also have significant financial impacts on the healthcare system.

In addition, patients and their families can potentially face significant financial impacts due to delayed reperfusion. For instance, a cross-sectional study conducted in Sri Lanka revealed that 40% of MI survivors who did not receive reperfusion therapy had to seek financial assistance for their out-of-pocket expenses.^19^Furthermore, the study discovered that 5% of patients lost their jobs; 29% had limited physical activity while remaining employed; 40% had employment time constraints; 15.4% applied for loans; 7.8% sold their property; 19.1% experienced an income loss; and 33.8% had to reduce their usual expenses.^19^In Brazil, 38% of cardiovascular deaths occur in working-age individuals, which causes a productive loss equivalent to 15% of the total cost associated with cardiovascular disease.^14^

Indeed, cardiovascular disease is particularly concerning in low and middle-income countries (LMIC), where it is estimated that 80% of all cardiovascular deaths occur.^20^In these countries, STEMI tends to affect younger working-age individuals, resulting in significant direct and indirect economic consequences. Furthermore, projections suggest that the cumulative economic loss from cardiovascular diseases in LMIC, between 2011 and 2025, will reach approximately US$3.76 trillion.^20^

Although LVEF, Killip class, and troponin levels were similar between the groups according to the time from symptom onset to thrombolysis in STEMI, individuals treated after 9 hours required rescue PCI more frequently, which was a major driver of worse outcomes. Additionally, because rescue PCI typically necessitates a time-consuming hospital transfer, individuals treated after 9 hours also had lower myocardial blush and TIMI flow grades, leading to increased use of glycoprotein IIb/IIIa inhibitors, adenosine, and dobutamine ( Table 1 ). These findings may underlie the occurrence of clinical endpoints such as stroke, major bleeding, and cardiac arrest, which may have contributed to the higher death rate in individuals treated later. Therefore, given that LVEF and troponin were similar across groups, it is more likely that the deaths were caused by non-cardiac or indirect factors.

Altogether, these findings underscore the need for well-planned strategies to facilitate faster reperfusion therapy. These strategies may include improvements in the transportation and triage of patients, implementation of effective treatment protocols, and utilization of advanced technologies to streamline the process of reperfusion therapy. By developing and implementing such strategies, healthcare providers can ensure that patients receive prompt and effective treatment, which can lead to better outcomes and lower costs.

In interpreting our findings, it is important to acknowledge certain limitations. While we were able to illustrate the cost implications of delayed reperfusion therapy, our study lacked the statistical power to estimate the cost per hour of delay. Moreover, we lacked access to some data concerning the wide range of financial impacts that can transpire within the families of patients with STEMI, which would have enabled us to generate a more comprehensive estimate of the total cost associated with delayed reperfusion. While the results were consistent throughout the entire study cohort, the limited sample size of patients who received pharmacoinvasive therapy rendered the cost analysis unreliable in this particular group of patients. It is also noteworthy that our methods for estimating indirect costs have a limitation as we were unable to capture long-term major clinical disability or clinical events occurring after discharge; thus, it relies only on in-hospital deaths.

## Conclusion

To sum up, our results provide further evidence supporting the critical role of prompt reperfusion therapy in preserving both lives and public health resources. The absence of a well-functioning system for managing STEMI cases generates both direct financial costs, such as in-hospital expenses, and indirect costs associated with the loss of productive years due to premature death or reduced working capacity.

## Data Availability

All requests for raw and analyzed data and related materials, excluding programming codes, will be reviewed by the Clarity Healthcare Intelligence legal department to verify whether the request is subject to any intellectual property or confidentiality obligations. Requests for patient-related data can be considered upon request. Any data and materials that can be shared will be released via a Material Transfer Agreement.

## *Supplemental Materials

For additional information Supplemental Material 1, please click here. https://abccardiol.org/supplementary-material/2024/12105/2023-0650_supplementary_material.pdf

For additional information Supplemental Material 2, please click here. https://abccardiol.org/supplementary-material/2024/12105/2023-0650_supplementary_checklist.pdf
